# The Aging Behavior and Life Prediction of CFRP Rods under a Hygrothermal Environment

**DOI:** 10.3390/polym15112490

**Published:** 2023-05-28

**Authors:** Xiaodong Liu, Qingyong Su, Jing Zhu, Xiaopeng Song

**Affiliations:** 1 School of Energy and Built Environment, Guilin University of Aerospace Technology, Guilin 541004, China; lxd@guat.edu.cn (X.L.); sqy@guat.edu.cn (Q.S.); 2 College of Civil Engineering and Architecture, Harbin University of Science and Technology, Harbin 150080, China

**Keywords:** hygrothermal aging, water absorption, thermal and mechanical performances, degradation mechanism, life prediction

## Abstract

Carbon fiber-reinforced polymer (CFRP) composites have been widely used in civil engineering structures due to their excellent mechanical and durability properties. The harsh service environment of civil engineering leads to significant degradation of the thermal and mechanical performances of CFRP, which then reduces its service reliability, service safety, and life. Research on the durability of CFRP is urgently needed to understand the long-term performance degradation mechanism. In the present study, the hygrothermal aging behavior of CFRP rods was investigated experimentally through immersion in distilled water for 360 days. The water absorption and diffusion behavior, the evolution rules of short beam shear strength (SBSS), and dynamic thermal mechanical properties were obtained to investigate the hygrothermal resistance of CFRP rods. The research results show that the water absorption behavior conforms to Fick’s model. The ingression of water molecules leads to a significant decrease in SBSS and glass transition temperature (Tg). This is attributed to the plasticization effect of the resin matrix and interfacial debonding. Furthermore, the Arrhenius equation was used to predict the long-term life of SBSS in the actual service environment based on the time–temperature equivalence theory, obtaining a stable strength retention of SBSS of 72.78%, which was meaningful to provide a design guideline for the long-term durability of CFRP rods.

## 1. Introduction

Fiber-reinforced epoxy resin-based (FRP) composites have been successfully applied in the aerospace field since the 1940s due to their excellent properties, such as light weight, high strength, fatigue resistance, and corrosion resistance [1,2,3]. With the development of FRP composite production technology, the performance of FRP composites is constantly improved, and the use price is also constantly reduced. Since then, FRP has been widely used in automotive, energy, and sports fields [4,5]. In order to solve the problem of steel bar corrosion, FRP has been widely used and applied in the field of civil engineering in recent years [6,7]. Since the 1980s, carbon fiber-reinforced epoxy resin-based (CFRP) composites were first applied in civil engineering structure strengthening [8,9]. CFRP has played a great role in the rapid repair of bridges and building structures after the Hanshin earthquake in Japan in 1995, and since then CFRP composites have been widely used in the reinforcement and repair of civil engineering structures. In recent years, CFRP composites have been applied to some new structures, such as bridge cable structures, prestressed reinforced concrete structures, etc. [10,11,12,13], which provide innovative development of civil engineering structures.

Civil engineering has the characteristics of a long service life and a harsh service environment compared with aviation and aerospace engineering [14,15], which inevitable leads to the performance degradation of CFRP under the reaction of humidity, heat, acid, alkali, and loading [16,17,18]. Among them, temperature and humidity have the greatest influence on the properties of composite materials [19,20,21]. The ingressions of water molecules will interact with the resin matrix, which leads to the performance degradation of composites. In addition, high temperatures will accelerate the degradation rate of CFRP composites [7,8]. Thus, the service reliability, safety, and life of CFRP composites and structures can be reduced. Relevant scholars have studied the durability of CFRP earlier, including temperature, humidity, ultraviolet light, alkali solution, salt solution, freeze-thaw cycle, and other influencing factors [22,23,24]. After aging under different conditions, the degradation behavior of the mechanical properties of CFRP composites is obtained, which provides design parameters for engineering applications [9]. Since the main factor causing the degradation of CFRP performance is the ingression of water molecules, it is necessary to carry out systematic research on the water absorption and diffusion behavior of CFRP in various solutions before studying the long-term performance evolution of CFRP [10,11].

When the CFRP composite is exposed to a hygrothermal environment, water molecules enter the inside of the CFRP rod through diffusion, the whole process being similar to the process of heat conduction. A large number of studies have shown that the water absorption behavior of CFRP composites generally conforms to Fick’s diffusion model [17,20,25]. Fick’s water diffusion model means that the quality of composite materials does not change after the composites reach water absorption saturation; meanwhile, the water absorption of composite materials reaches quasi-dynamic equilibrium. When thewater absorption curve deviated from Fick’s model, some scholars proposed non-Fick’s diffusion models [21,26], such as the two-stage diffusion model and Langmuir’s diffusion model. The debonding of the fiber-resin interface provides more channels for the further ingression of water molecules, which led to the non-Fick’s diffusion behavior.

Long-term hygrothermal aging brought about the inevitable degradation of thermal and mechanical performances for CFRP [27,28,29,30]. Owing to the mechanical properties of carbon fiber, they are not affected by water absorption. Therefore, the mechanical degradation of composite materials mainly comes from the interface and resin matrix. When water molecules ingress into the CFRP rod, part of the water molecules exists in the hole and free volume of matrix, which forms the free water. The hygrothermal expansion effect caused by free water will lead to the cracking of the resin matrix and interfacial debonding. Some of the water molecules interact with the resin matrix, which forms the bonding water. The bonding water will replace the hydrogen bond of molecular chains, which would increase the distance of molecular chains significantly and the movement between molecular chains [31,32]. It is well known that glass transition temperature (Tg) is an important parameter of composites, and Tg determines the maximum usage temperature of composites in engineering applications. The variation of Tg can reflect the interchain structure and the degree of resin plasticization occurring in the material [33,34]. Understanding the degradation mechanisms of Tg and the prediction of Tg are critical for material applications in a hygrothermal environment. In addition, the bonding water can also break the hydrogen bond and van der Waals force between fiber and resin, which leads to interfacial debonding. The interfacial strength is one of the key factors in the service performance of CFRP composites. The degradation of the interface can weaken the coordination between fiber bundles, which results in the degradation of the tensile strength.

Some researchers [35,36,37] investigated the long-term durability of FRP under corrosion environments (artificial salt water, seawater and sea sand concrete environments, and marine-based environments) and obtained the degradation law and mechanism of long-term properties. However, they did not systematically study the diffusion behavior of water molecules in FRP and did not reveal the thermal and mechanical property degradation mechanisms caused by the ingression of water molecules. Therefore, it is necessary to further investigate the water absorption and diffusion behavior and the thermal and mechanical evolution of CFRP rods. In the present study, CFRP rods were exposed to distilled water at 40 °C, 60 °C, and 80 °C for 360 days. The water absorption behavior, thermal properties, and mechanical property evolution were obtained through experiments. The degradation of the interface between fiber and resin was observed by scanning electron microscopy. Based on the time–temperature equivalence theory, the Arrhenius formula was used to predict the long-term life of SBSS in the actual service environment, which was meaningful for providing the design guidelines for civil engineering structures.

## 2. Experimental Procedures

### 2.1. Raw Materials

**Figure 1:**
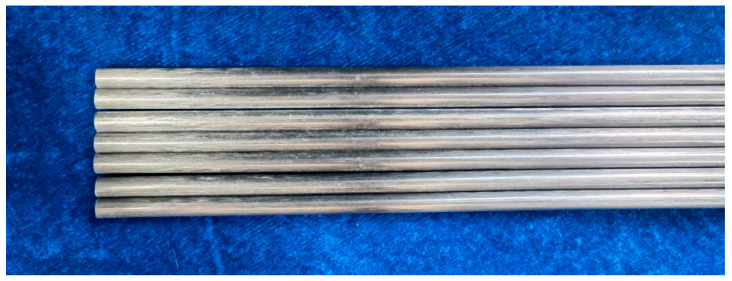
CFRP rods used in the experiment.

A CFRP rod with a 7 mm diameter produced by pultrusion technology was used in the current research, as shown in Figure 1. T700 carbon fiber (12 K) and bisphenol-A epoxy resin (Blue Cube Chemicals Company, China) were used for the production of CFRP rods, and the detailed mechanical performances of the fiber and matrix are listed in Table 1. In addition, the volume fraction of fiber was about 70%.

### 2.2. Water Absorption and Desorption Test

The water absorption test of CFRP rods was carried out according to ASTM D5229M-14. When the length of the rod is greater than 30 times its diameter, it can be considered that the water mainly ingresses along the rod radially, and the ingression along both ends of the rod can be ignored [16]. To be conservative, CFRP rods were cut to 240 mm in length in the present paper. Before the water absorption test, all samples were dried in a 60 °C oven for one week to remove the original residual moisture. The specimens were exposed to 40 °C, 60 °C, and 80 °C in deionized water, in reference to other research work [38,39]. An electronic balance (Shanghai Jingke Industry Co., Ltd., Shanghai, China) with an accuracy of 0.1 mg is used to measure the mass change of water absorption periodically, and five specimens were repeated in each condition. After the measuring, the specimens were rapidly returned to the water bath. Five specimens were tested for each condition, and the mass change percentage of water absorption for each specimen was calculated using the following equation:

(1) $$M_{t}=\frac{W_{t}-W_{0}}{W_{0}}\times 100$$

where *M_t_* is the percentage of water absorption; *W*_0_ is initial specimen weight; and *W_t_* is the exposed specimen weight.

The same specimen dimensions were used in the water desorption tests. The specimens, after exposure for 360 days, were dried in an oven at 60 °C for 55 days and then at 120 °C for 35 days until they reached the unchanged weight. Five specimens were tested for each condition. The mass changes of water desorption after drying for each specimen are calculated using the following equation:

(2) $$M_{dt}=\frac{W_{dt}-W_{0}}{W_{0}}\times 100$$

where *M_dt_* is the percentage of water desorption and *W_dt_* is the dried specimen weight.

### 2.3. Short Beam Shear Strength Test

**Figure 2:**
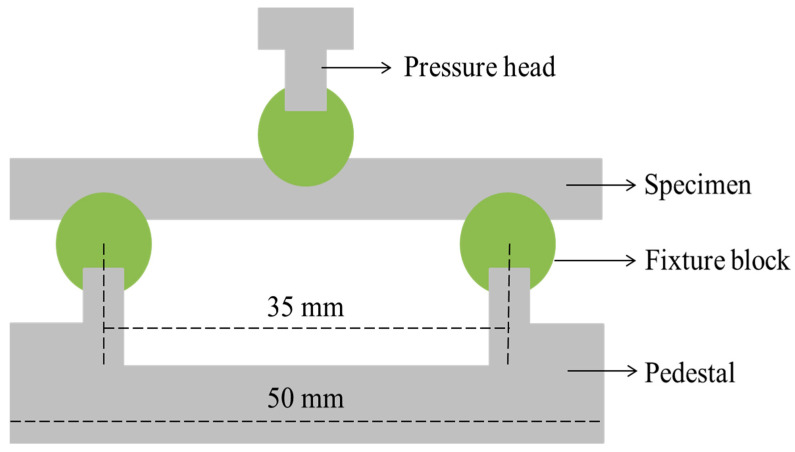
The short beam shear test device diagram of a CFRP rod.

The water molecules will react with the polar resin matrix, resulting in the degradation of the interfacial properties. The short beam shear test can well characterize the interfacial strength of the fiber and resin matrix. The short beam shear test of CFRP rods was carried out according to ASTM D4475. CFRP rod was cut into 50 mm as short beam shear test samples, and the span was 35 mm (five times the diameter of the rod). A short beam shear test was carried out with a universal machine (LE5105, Shanghai, China), and the loading speed was set to 1.3 mm/min. The detailed short beam shear test device diagram of CFRP rods is shown in Figure 2. The aging specimens exposed to water were carried out the short beam shear test at periodic intervals of 30 days, 90 days, 180 days, and 360 days. Each condition tested five samples to obtain the average.

### 2.4. Dynamic Thermal Mechanical Analysis (DMA)

The reaction between water and resin will inevitably change the cross-linking density of the matrix, which can be characterized by DMA. In addition, the maximum service temperature of CFRP rods is usually related to T_g_, and the degraded cross-linking density can reduce T_g_. Therefore, a DMA test is necessary to carry out to obtain the evolution of dynamic thermal mechanical properties after hygrothermal aging. DMA tests of CFRP rods were carried out according to ASTM E1640-9. The specimens were cut into rectangles with a size of 35 mm × 5 mm × 2 mm. The actual dimensions of each specimen were measured using a micrometer with an accuracy of ±0.001 mm. The aged specimens were subjected to the DMA tests through the dynamic mechanical analyzer (Q800, TA Instruments, America) at periodic intervals of 30 days, 90 days, 180 days, and 360 days. The single cantilever beam clamp mode was selected with a load frequency of 1 Hz, an amplitude of 20 μm, and a heating rate of 5 °C/min from room temperature to 250 °C. Each condition tested two samples to obtain the average of T_g_.

### 2.5. Scanning Electron Microscopy

The degradation of thermal and mechanical properties was related to the microscopic damage mechanism of CFRP rods. In order to figure out the detailed reason, scanning electron microscopy (SEM) tests were performed to characterize the microstructure damage. After exposure for 360 days, the fracture morphology of short beam shear specimens was observed through the scanning electron microscope (SEM, VEGA3, Czech TESCAN, Brno, and Czech). Before the tests, the samples were vacuumed and sprayed with gold to increase their electrical conductivity. The frequency of 1000 Hz, 0.7 A current, and 30 kV voltage amplitude were selected in the test.

## 3. Results and Discussion

### 3.1. Water Absorption and Desorption

**Figure 3:**
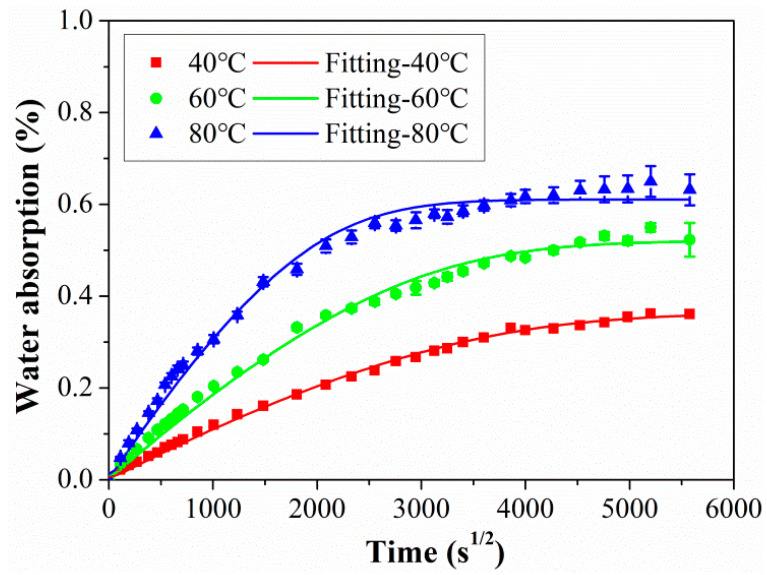
The water absorption curves of rod exposure at 40 °C, 60 °C, and 80 °C.

CFRP rods were immersed in distilled water, and the quality of the rods was tested periodically. The water absorption behaviors of CFRP rod exposure at 40 °C, 60 °C, and 80 °C are shown in Figure 3. It can be seen that the water absorption of CFRP rods increased linearly with the immersion time in the initial stage, and then the water absorption rate gradually slowed down and tended to be stable. It was obvious that the water absorption of the CFRP rod conformed to classical Fick’s diffusion behavior. Combined with diffusion mathematics, Equation (3) can be used to fit the water absorption test data of the CFRP rod, and the fitting results are shown as the solid line in Figure 3.

(3) $$M_{t}=M_{\infty }\left[1-\sum_{n=1}^{\infty }\frac{4}{R^{2}\alpha_{n}^{2}}\exp(-D\alpha_{n}^{2}t)\right]$$

where *M*_∞_ is the percentage of pseudo-equilibrium water absorption; *D* is the diffusion coefficient; *R* is the diameter of the CFRP rod; and *α_n_* is the nth root of Bessel function of order zero.

**Figure 4:**
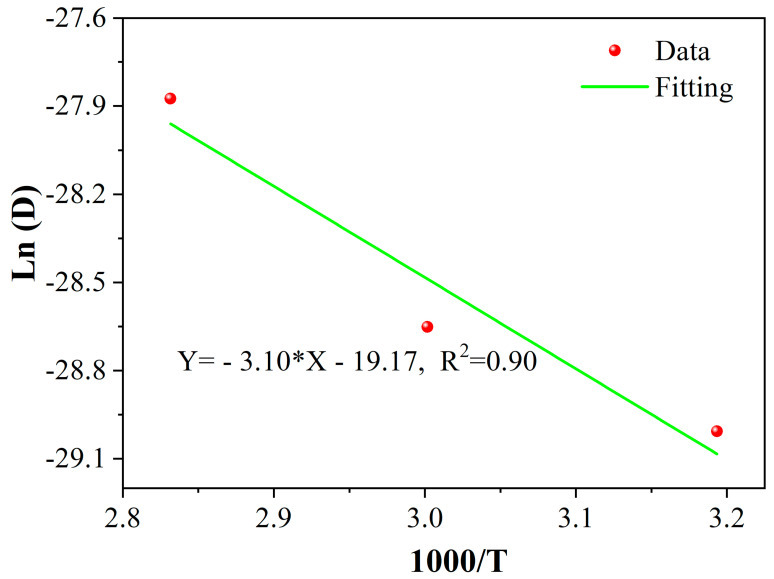
The linear relationship of ln (*D*) and 1000/T.

As shown in Figure 3, the Fick’s diffusion model of Equation (3) fitted very well with the experimental data, and the detailed fitted parameters of *M*_∞_ and *D* were listed in Table 2. As found, the parameters *M*_∞_ and *D* were highly dependent on the exposure temperature. This was because the elevated temperature speeded up the diffusion rate of water molecules, which brought about a higher value of *D* at higher temperatures. In addition, the elevated temperature can intensify the segment motion of the resin matrix, which increases the free volume of the matrix for the ingression of water molecules, leading to a higher value of *M*_∞_. Furthermore, according to the classic Arrhenius equation, as shown in Equation (4) [17], the linear relationship between Ln (*D*) and 1/T was established and shown in Figure 4.

(4) $$D=D_{0}\exp(-\frac{E_{a}}{RT})$$

where *E*_a_ is the activation energy of the water diffusion process, *R* is the universal gas constant, and *D*_0_ is constant. By fitting, the *E*_a_ of the resin is 25.77 kJ/mol. It can be seen that the linear fitting line fitted very well with the experimental data, which proved that the water absorption of CFRP rods satisfied the temperature acceleration of Arrhenius.

To further figure out the diffusion behavior of water molecules in CFRP rods, the normalized concentration distribution of water molecules along the radial direction of the rod was obtained quantitatively by diffusion mathematical theory. Obtaining the diffusion process of water molecules along the radial direction for a long cylinder according to Equation (5). It can be seen that the water concentration was a function of the radial position *r* and time *t*.

(5) $$\frac{\partial C(r,t)}{\partial t}=\frac{1}{r}\frac{\partial }{\partial t}(rD_{⊥}\frac{\partial C(r,t)}{\partial r})$$

**Figure 5:**
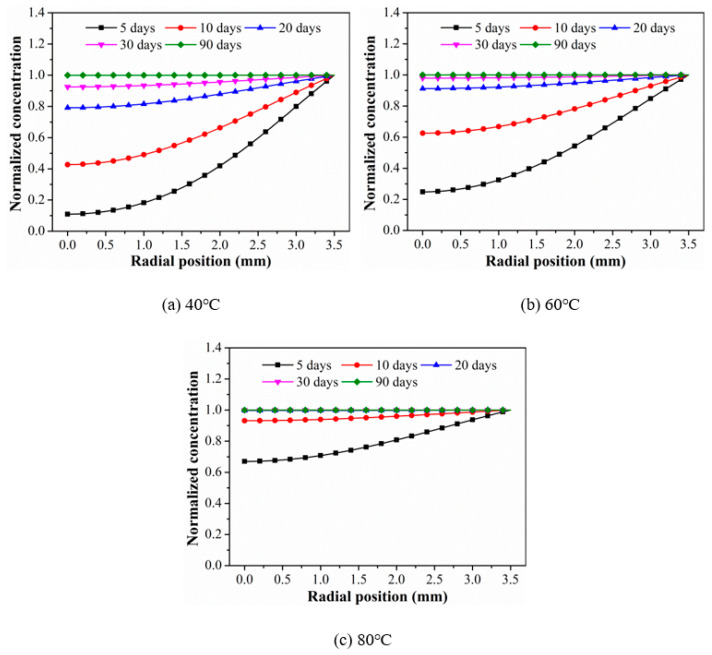
The variations in normalized concentration of CFRP rods with radial positions at specific exposure times.

The variations in normalized concentration of CFRP rods with radial positions at the time of 5, 10, 20, 30, and 90 days are shown in Figure 5. As found, the normalized concentration increased with time at the same exposure temperature and radial position. In addition, the normalized concentration increased with the increase in radial position. The exposure temperature had an important effect on the normalized concentration. It was obvious that the elevated temperature increased the normalized concentration significantly, which reduced the time of the core until the CFRP rod reached saturation concentration. This was because the elevated temperature speeded up the diffusion rate of water molecules along the radial direction of the CFRP rod.

**Figure 6:**
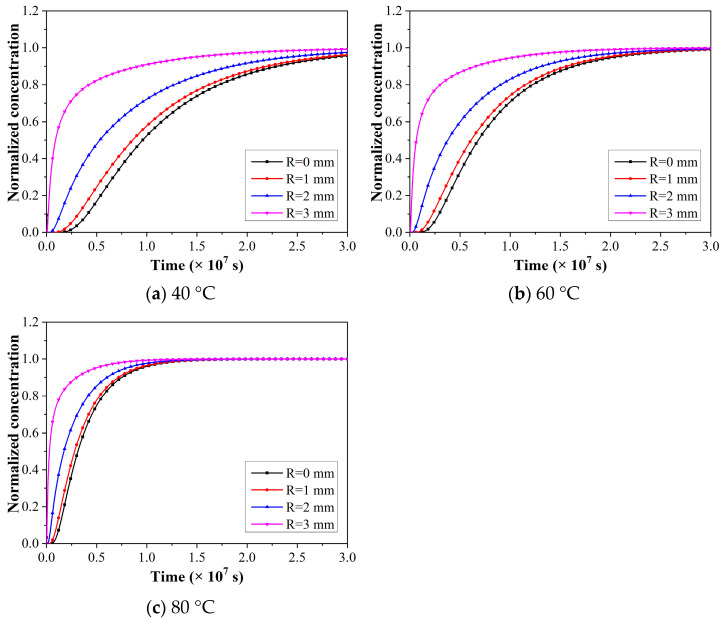
The variations of normalized concentrations of CFRP rods with exposure times at specific radial positions.

The variations of the normalized concentration of CFRP rods with exposure time at a specific radial position are shown in Figure 6. As found, the curves of normalized concentration increased quickly first at the initial exposure temperature, and then the normalized concentration increased at a slower rate and gradually trended to be stable. In addition, the normalized concentration increased significantly with radial position at the same exposure temperature and time, and the elevated temperature reduced the time that reached saturation concentrations.

The normalized water concentration of 0.99 was selected as the saturated state of water absorption, and the corresponding time was the saturated water absorption time, as shown in Table 3. It was obvious that the elevated temperature reduced the time it took to reach saturated water absorption significantly. Such as, for the radial position of 3 mm, the times reached for saturated water absorption were 33.4, 23.5, and 10.8 days for 40 °C, 60 °C, and 80 °C, respectively. In conclusion, temperature was the main factor affecting the water absorption and diffusion behavior in the rods.

**Figure 7:**
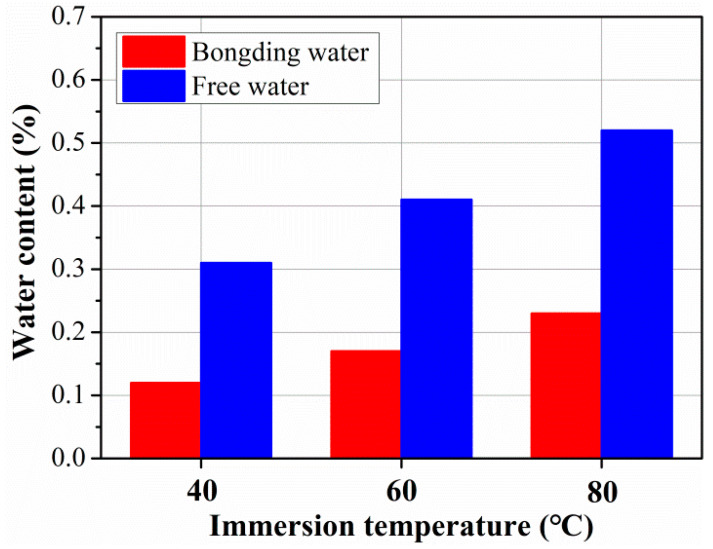
The content of free water and bonding water after 360 days of rod exposure at different temperatures.

**Figure 8:**
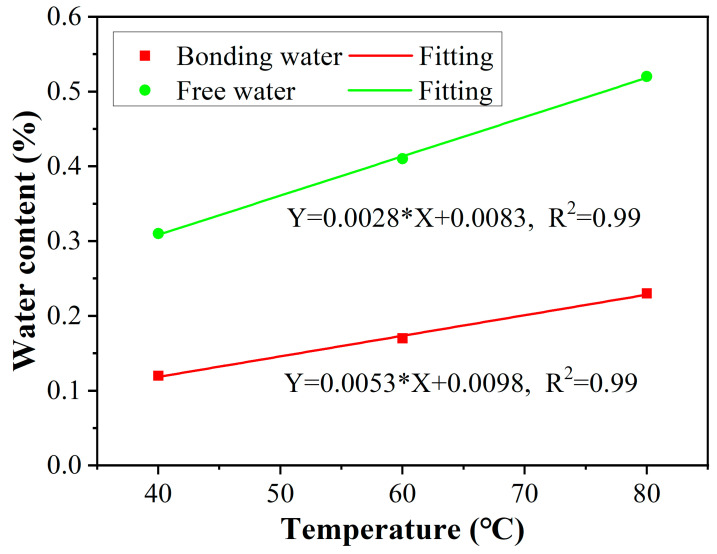
The relationships between free water content, bonding water content, and exposure temperature.

The content of free water and bound water in the rod after 360 days of immersion was obtained by water desorption tests in reference to the research of [31], as shown in Figure 7. As found, the content of free water was higher than that of bonding water, and the detailed contents of free and bonding water were 0.12 and 0.31 for 40 °C, 0.17 and 0.41 for 60 °C, and 0.23 and 0.52 for 80 °C. It is obvious that high soaking temperatures increase the content of free water and bound water, which is because the elevated temperature accelerates the diffusion of free water and the form of bonding water. In addition, in order to figure out the quantitative effect of temperature on the water contents, the linear relationship between free water content, bonding water content, and exposure temperature was established, as shown in Figure 8. As found, the content of free and bonding water increased linearly with exposure temperature; the slope of free water was higher than that of bonding water, which is attributed to the performance of bound water being more difficult than that of free water.

### 3.2. The Degradation of Short Beam Shear Properties

**Figure 9:**
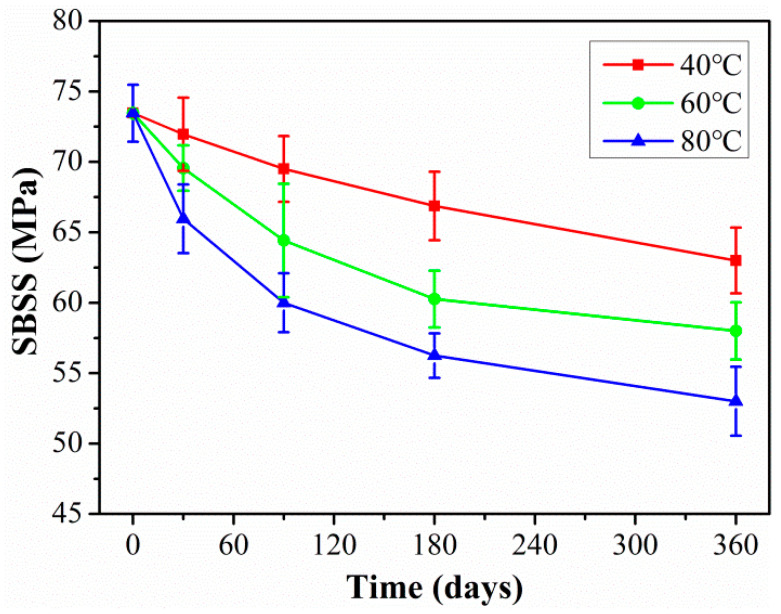
The changes in SBSS with exposure time of rods exposed at different temperatures.

The changes in SBSS with the exposure time of rod exposure at different temperatures are shown in Figure 9. As shown in Figure 9, the shear strength degradation rate was relatively fast in the initial exposure stage and then gradually slowed down. This was mainly because a lot of water molecules ingressed into the CFRP rod in the initial phase, and the water molecules broke up the van der Waals force and hydrogen bond between the fiber and resin matrix, which led to significant degradation of the interface. As the water absorption of the rod approaches saturation, the degradation effect of water molecules on the interface gradually weakens. At the same exposure time, high temperatures will significantly accelerate the decrease in shear strength. It can be seen that the exposure temperature was the main factor affecting the interfacial strength of the CFRP rod.

**Figure 10:**
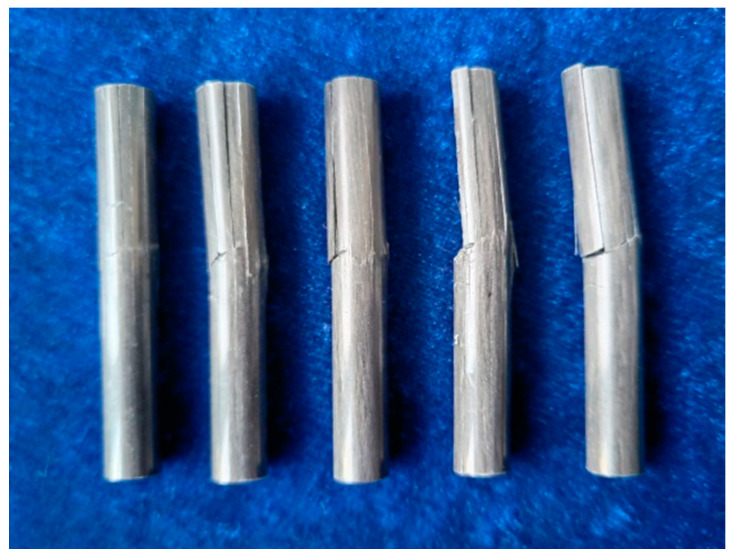
The failure mode of the short beam shear of CFRP rods.

The failure mode of short beam shear of CFRP rods is shown in Figure 10. As found, the typical interlayer shear failure occurred in the short beam shear tests, and the interlayer failure occurred on one side of the span. As can be seen from the cross-sectional failure diagram, the shear cracks on the interface are distributed from the middle to both sides. This is mainly because shear failure occurs first in the neutral axis, where the shear stress is higher, and then spreads to both sides. It should be noted that both the control and the aged rods had the same shear failure mode, and the effect of temperature on the shear failure mode was negligible.

### 3.3. The Degradation of Dynamic Thermal Mechanical Properties

**Figure 11:**
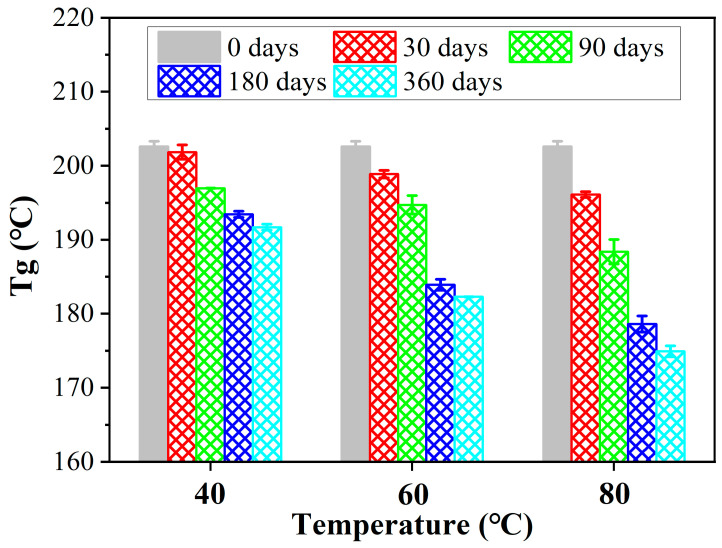
The changes in T_g_ of rod exposure at 40 °C, 60 °C, and 80 °C with exposure time.

The changes in T_g_ rods exposure at 40 °C, 60 °C, and 80 °C are shown in Figure 11. As found, the hygrothermal environment had a significant effect on the T_g_. At the same temperature, T_g_ continued to decrease with exposure time, which was mainly due to the plasticization of the resin matrix caused by the ingression of water molecules. The ingression of water molecules replaced the hydrogen bonds between molecular chains and increased the freedom of molecular chains, which significantly improved the movement of molecular chains. Similar to the degradation of SBSS, the degradation rate of T_g_ slowed down as water absorption approached saturation. At the same exposure time, T_g_ decreased significantly with the exposure temperature. It can be concluded that the exposure temperature was also the key factor of degradation for T_g_ because the higher temperature sped up the ingression rate of water molecules and caused more reaction with the resin matrix.

**Figure 12:**
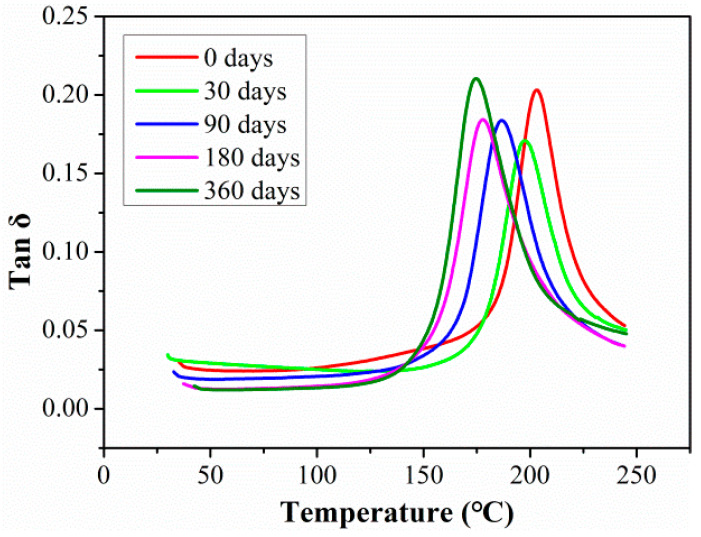
The changes in tanδ curves with aging time of rod exposure at 80 °C.

The changes in tanδ curves with the aging time of rods exposure to 80 °C are shown in Figure 12. The curve peak of Tan delta decreased in the initial exposure stage of 30 days, and then the curve peak of Tan delta increased with exposure time. Which was because the plasticization of the matrix reduced the crosslinking density between the molecular chains at the initial stage, woken the damping characteristics of the resin matrix, and then reduced the curve peak of Tan delta. With the ingression of a large number of water molecules into CFRP rod, the fiber-resin interface had significant debonding, and the limitation of the interface on the molecular chain of resin matrix was reduced, which greatly improved the movement freedom of the molecular chain and the damping performance of resin matrix. In addition, the abscissa of the peak value of Tan delta shifted to the left with the exposure time, which implied the degradation of T_g_.

**Figure 13:**
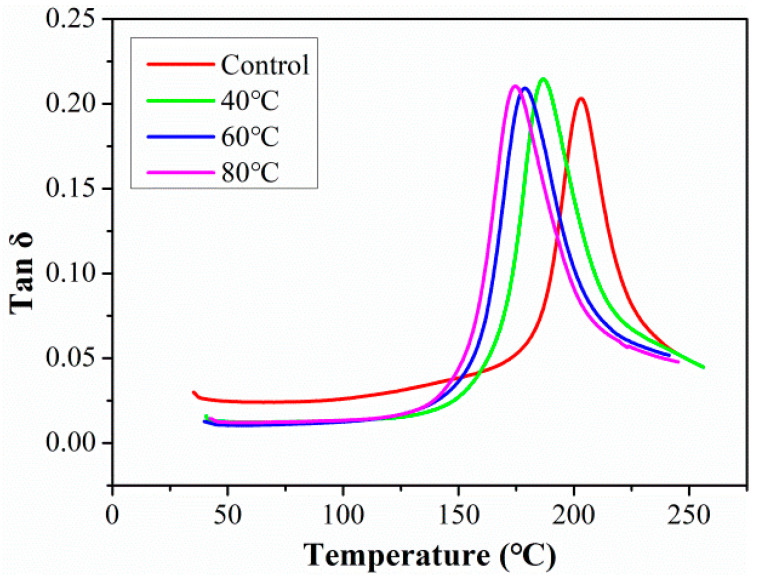
The changes in tanδ curves with aging temperature of the rods after 360 days.

The changes in tanδ curves with the aging temperature of rods after the exposure of 360 days are shown in Figure 13. As found, the exposure temperature had no significant effect on the curve of the peak of the Tan delta. This was because after the exposure of 360 days, the CFRP rods at all immersion temperatures reached water absorption saturation, and the interface was severely degraded. In addition, the shift to the left of the abscissa for the peak value of the Tan delta increased with exposure temperature, which showed again that the effect of temperature on the T_g_ was significant.

### 3.4. Microscopy

**Figure 14:**
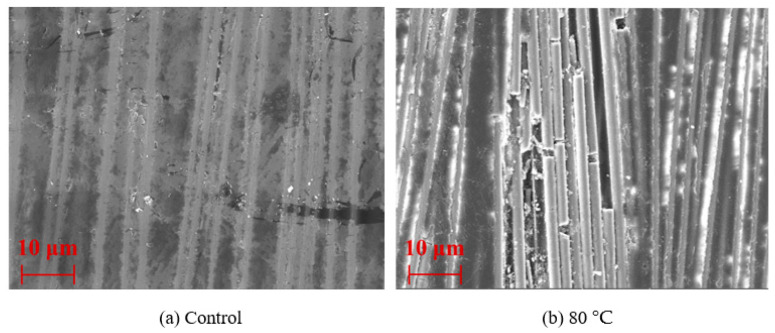
The surface topography of the CFRP rods of (**a**) control and (**b**) aged specimens.

The surface topography of CFRP rods from control and aged specimens is shown in Figure 14. As shown in Figure 14a, the surface of the CFRP rod was smooth, the carbon fiber was tightly wrapped by the resin matrix, and there was no obvious defect on the surface. On the contrary, the resin on the surface of the rods peeled off, and the carbon fibers were exposed after the rods were immersed in water at 80 °C for 360 days. This was because higher exposure temperatures led to the degradation of the resin on the surface of the rods.

**Figure 15:**
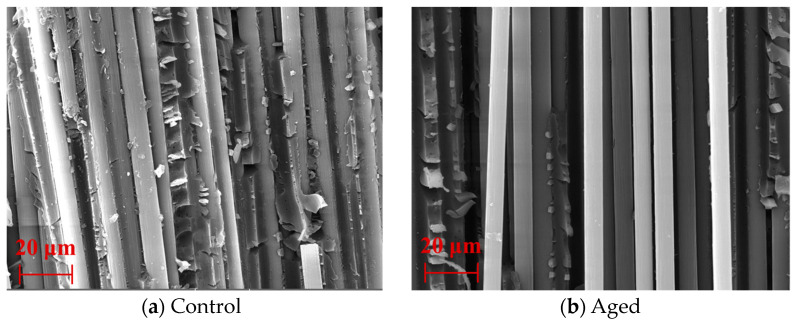
The fracture topography of short beam shear for control and aged specimens.

The fracture topography of short beam shear in control and aged specimens is shown in Figure 15. As found, a large amount of resin remained on the surface of the carbon fiber in the control specimens, and the fiber is tightly wrapped by the resin matrix. The whole shear fracture topography was relatively complete, and the resin showed ductile shear failure. By contrast, no resin remained on the surface of the carbon fiber after the hygrothermal aging at 80 °C for 360 days, and the fiber distribution became messy throughout the shear fracture. This indicated that the ingression of water molecules caused serious interfacial debonding between the fiber and resin matrix.

### 3.5. The Long-Term Life Prediction of Short Beam Shear Properties

#### 3.5.1. Arrhenius Theory

The Arrhenius equation is an empirical formula for the relationship between the rate constant of chemical reactions and temperature created by Arrhenius of Sweden, as shown in Equation (6).

(6) $$k=A\exp(-E_{a}/RT)$$

where *k* is the performance degradation rate; *A* is a constant; *E*_a_ is the activation energy; *R* is the universal gas constant; and *T* is the absolute temperature.

According to Equation (6), Arrhenius assumed that the increase in temperature will speed up the chemical reaction rate under the premise that the chemical reaction mechanism was unchanged. The time required for the same chemical reaction at different temperatures satisfied the following relationship:

(7) $$TSF=\frac{t_{0}}{t_{1}}=\frac{k_{1}}{k_{0}}=\frac{A\;\exp(-E_{a}/RT_{1})}{A\exp(-E_{a}/RT_{0})}=\exp\left[\frac{E_{a}}{R}(\frac{1}{T_{0}}-\frac{1}{T_{1}})\right]$$

where *t*_0_ and *t*_1_ are the required times under the temperatures of *T*_0_ and *T*_1_. The ratio *t*_0_/*t*_1_ is the time-shift factor *TSF*.

#### 3.5.2. Life Prediction of SBSS

Based on the Arrhenius equation, a large number of scholars have proposed prediction models for the interfacial strength degradation of FRP composites [40,41]. Phani et al. [42] believed that the interfacial properties of FRP composites will decrease to a stable level and converge to a constant with the increase in aging time. The detailed degradation model between interfacial strength and aging time is shown in Equation (8).

(8) $$Y_{t}=\left(100-Y_{\infty }\right)\exp(-t/\tau )+Y_{\infty }$$

where *Y_t_* is the retention; *t* is the exposed time; *τ* is the fitted parameter; and *Y*_∞_ is the final strength retention.

**Figure 16:**
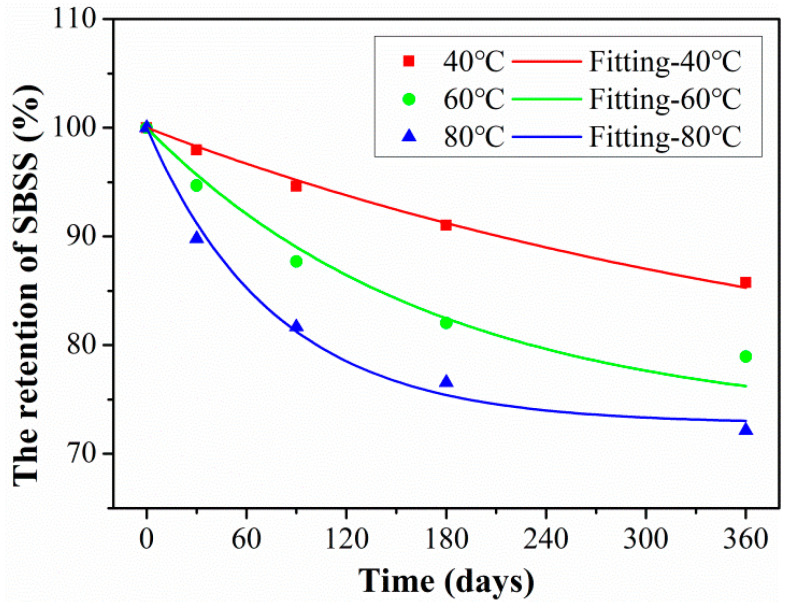
Long-term prediction curves of SBSS of rod exposure at 40 °C, 60 °C, and 80 °C.

According to the related research [40,41], Equation (8) was used to nonlinear fit SBSS in Figure 9. The fitted curves of SBSS of rod exposure at 40 °C, 60 °C, and 80 °C were obtained as shown in Figure 16, and the detailed fitted parameters were listed in Table 4. As found, it fitted well between the fitting curve and the experimental data, which indicated that the degradation of SBSS satisfied the Arrhenius acceleration theory. The fitted parameter *τ* decreased with exposure temperature; this indicated the elevated temperature speeded up the degradation rate of SBSS.

In order to obtain *TSF* between the laboratory aging temperature and the actual service temperature, the activation energy *E*_a_ of SBSS degradation should be obtained first. Furthermore, by transforming Equation (6) into Equation (9), and then taking the fitted parameters *τ* and *Y*_∞_ into Equation (9), the time *t* can be obtained when SBSS reached 84%, 88%, 92%, and 96% retention at 40 °C, 60 °C, and 80 °C, respectively.

(9) $$\ln\left(\frac{1}{k}\right)=\frac{E_{a}}{R}\frac{1}{T}-\ln A$$

**Figure 17:**
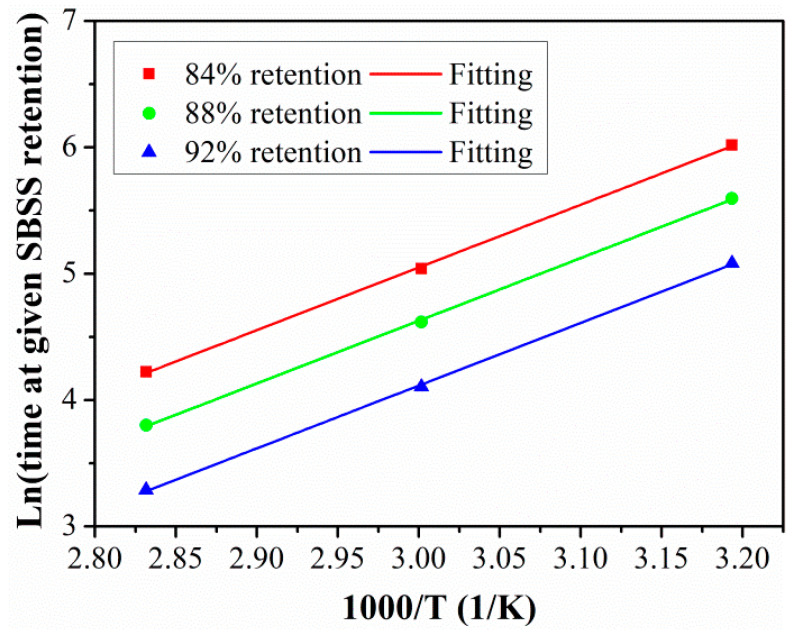
Arrhenius plots of SBSS retention.

According to Equation (9), plot the scatter points of Ln (1/*k*) and 1000/T. The linear fitting was used to establish the relationship between the time (1/*k*) and 1/T. As shown in Figure 17, the fitting curves fitted well with scatter points, and the fitting curves were parallel with each other; this also proved that the degradation of SBSS under a hygrothermal environment satisfied the Arrhenius theory. In addition, the detailed fitted parameters of *Ea*/*R* are listed in Table 5. It should be noted that the slope of *Ea*/*R* represented the energy barrier of SBSS degradation, and the higher value of *Ea*/*R* represented the low degradation rate of SBSS.

By substituting *Ea*/*R* in Table 5 into Equation (7), the *TSF* between the laboratory aging temperature and the actual service temperature can be calculated. In the present paper, three service temperatures of 8.1 °C, 15.8 °C, and 21.8 °C were selected referencing the research of [40] to conduct the long-term life prediction of SBSS, and the calculated *TSF* is listed in Table 6.

**Figure 18:**
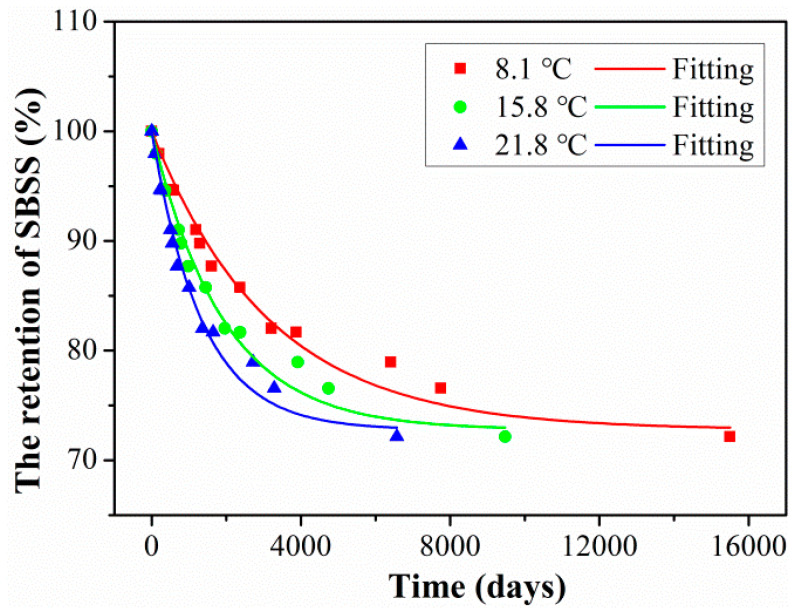
Long-term life decrease curves of SBSS of rods at three actual service temperatures.

The exposure time (abscissa in Figure 9) in the laboratory multiplied by the time-shift factor (*TSF*) can obtain the required time at the actual service temperature for CFRP rod. Furthermore, the long-term life prediction curves can be obtained through the above prediction model of Equation (8), and the prediction results are shown in Figure 18. The detailed fitted parameters are listed in Table 7. As found in Figure 18, at the actual service temperature, the SBSS decreased rapidly at the initial aging stage and then tended to slow down. Higher exposure temperatures increased the degradation rate of SBSS in the initial stage; meanwhile, higher exposure temperatures decreased the time until SBSS reached stable retention. The long-term life retention of SBSS reached a stable level of 72.78%, which was meaningful to provide a design guideline for civil engineering structures.

In order to quantitatively analyze the degradation rate during the initial exposure, the required service time was obtained when SBSS reached 90% retention, and the detailed results are listed in Table 8. Furthermore, the service times when the retentions of SBSS decreased to 90% were 1442 days, 881 days, and 611 days for exposure temperatures of 8.1 °C, 15.8 °C, and 21.8 °C, respectively. It can be seen that the effect of service temperature on the initial degradation rate of SBSS was significant.

## 4. Conclusions

The diffusion behavior of water molecules in the composites and its effect on the long-term thermal and mechanical properties are still unclear. In order to ensure the service reliability and safety of CFRP rods under a hygrothermal environment, the water absorption behavior and thermal and mechanical degradation mechanisms were experimentally investigated. In addition, the Arrhenius equation was used to predict the long-term life of SBSS in the actual service environment based on the time–temperature equivalence theory. The following conclusions can be drawn based on the results and discussion:

(1) The water absorption of the CFRP rods conformed to the classical fickle diffusion model; high temperatures accelerated the diffusion rate of water molecules along the radial direction of the CFRP rod, which led to a higher diffusion coefficient of *D*.
(2) After the ingression of water molecules into CFRP rod, which interacted with the polar resin matrix and resulted in the plasticization of the resin matrix and the interfacial debonding of the fiber and resin matrix, which led to the significant degradation of the thermal and mechanical properties of CFRP rods.
(3) The falloff of the surface resin in the rod immersed in high-temperature water was found by scanning electron microscopy, and serious interfacial debonding occurred between the fiber and resin matrix.
(4) The Arrhenius equation was used to predict the long-term life of SBSS in the actual service environment based on the time–temperature equivalence theory, obtaining a stable strength retention of SBSS of 72.78%, which was meaningful to provide a design guideline for civil engineering structures.

## Figures and Tables

**Table 1:** The mechanical properties of fibers and resin.

Mechanical Properties	Carbon Fiber	Resin
Tensile modulus (GPa)	230	2.50
Tensile strength (MPa)	4900	112.5
Elongation (%)	2.13	4.50

**Table 2:** The fitting parameters of water absorption parameters for CFRP rods (*M*_∞_, *D*). *R*^2^ is the correlation coefficient used to characterize the fitting degree.

Temperature (°C)	Fitting Parameters		
	*M*_∞_ (%)	*D* (×10^−12^ m^2^/s)	*R* ^2^
40	0.36	2.50	0.99
60	0.52	3.56	0.99
80	0.61	7.74	0.99

**Table 3:** The time (days) required for saturated water absorption at different radial positions and exposure temperatures.

Exposure Temperature (°C)	Radial Position (mm)			
	R = 0	R = 1	R = 2	R = 3
40	49.79	48.61	44.48	33.44
60	34.97	34.13	31.25	23.51
80	16.11	15.69	14.38	10.80

**Table 4:** Fitted parameters of SBSS for CFRP rods.

Temperature (°C)	Fitted Parameters		
	*τ*	*Y*_∞_ (%)	*R* ^2^
40	463.15	72.78	0.99
60	174.24	72.78	0.97
80	77.09	72.78	0.99

**Table 5:** Fitted parameters of the Arrhenius plot of SBSS retention.

Retention (%)	*E*_a_ */R*	*R* ^2^
84	5198.15	0.99
88	5198.15	0.99
92	5198.15	0.99

**Table 6:** *TSF* of SBSS of rod exposure at actual service temperatures of 8.1 °C, 15.8 °C, and 21.8 °C.

Exposure Temperature (°C)	Service Temperature (°C)		
	8.1	15.8	21.8
40	6.57	4.01	2.78
60	17.79	10.87	7.54
80	43.05	26.31	18.24

**Table 7:** The fitted parameters of SBSS of rods at actual service temperatures.

Service Temperature (°C)	*τ* (1/d)	*R* ^2^
8.1	3150.12	0.99
15.8	1924.80	0.99
21.8	1334.95	0.99

**Table 8:** Required time when SBSS reaches 90% retention.

Service Temperature (°C)	Service Time (Days)
8.1	1442.38
15.8	881.33
21.8	611.25

## Data Availability

Not applicable.
